# Editorial: Human and Social Competition: An Interdisciplinary and Transdisciplinary Perspective

**DOI:** 10.3389/fpsyg.2019.02240

**Published:** 2019-10-01

**Authors:** Monica Thiel, Julia Levashina, Gabriele Giorgi, Aaron Williamon, Darren C. Treadway, Kai Wen, Qing Wang

**Affiliations:** ^1^University of International Business and Economics, School of Public Administration, Beijing, China; ^2^Kent State University, College of Business Administration, Kent, OH, United States; ^3^Department of Human Science, Universitá Europea di Roma, Rome, Italy; ^4^Centre for Performance Science, Royal College of Music, London, United Kingdom; ^5^Jacobs Management Center, University at Buffalo, Buffalo, NY, United States; ^6^College of Mechanical and Transportation Engineering, China University of Petroleum, Beijing, China

**Keywords:** human and social competition, organizational behavior (OB), strategic behavior, evolution behaviors, motivation, leadership style, attitude - behavior relationships, interactions

Organizational competition is a troublesome problem due to how competition is deeply embedded within human, social, relational, and organizational structures. The traditional views of competitiveness such as productivity, human and social capital, technology, social innovation, and socio-economic development lack adequate evaluation of human society's ability and capacity to shape and direct sustainable competitive advantage apart from government and business (Thiel, 2017). Moreover, it is a challenge to effectively identify and map human and social competition to organizational behavior and performance. For instance, some employees may secure their knowledge of a subject in the workplace as a way to demonstrate their social status and value over others through collective enactment. The characteristics of problems span from a focus on an organizational culture that does not address social structure linkages of human and social competition between the firm, within the firm and connections through stakeholder interactions. Further to this, it is an imperative to pay attention to human, social, and relational capital due to how these capitals shape and drive market and non-market performance. Current research on competition offers little insight of human and social competition in the firm beyond strategic positioning between firms in management, social comparison in social psychology, intra-personal competitiveness as competitive personality in psychology, and competition as game theory in economics (Swab and Johnson, 2018). Consequently, It is a challenge for scholars to address competition due to differing research traditions combined with varied perspectives, theories, and definitions of competition. Although increased business competition improves market competition, little research addresses the effects of human and social competition in the workplace and its impacts on organizational behavior. The lack of human and social competition in key debates of organizational psychology literature requires transitions to new opportunities for improving our understanding of human and social competition in the workplace.

Although Fletcher and Nusbaum (2010) provide five components of competition, namely competition for tangible rewards, non-tangible rewards, recognition, status, and the competitive influence of coworkers for measuring competition in workplace, the eight articles accepted and published in this Research Topic provide more detailed approaches and measurable concepts to expand and improve our understanding of human and social competition in organizations, including its impacts and opportunities for future research in organizational psychology. The first article in the Research Topic, authored by Wang et al. introduce two measurable concepts, namely “competitive attitude” and “competitive behavior.” The authors examine how these two concepts influence job crafting and job performance in employee personal characteristics and team climate. Wang et al. findings indicate both new concepts simultaneously influence job behavior in different ways. For example, trait competitiveness can be an antecedent of competitive attitude and competitive behavior. The second article in the collection proposes success in competitive settings are mainly driven by strategic ability. The authors, Bilancini et al. identify two main dimensions to test the reliability of strategic ability, namely mentalization (understanding of others' preferences and understanding of others' cognitive skills) and rationality (capacity to optimize as well as capacity to iterate reasoning) to move current and future research in human and social competition toward a measure of strategic ability. Articles three and four highlight the role of leadership in human and social competition. In article three, Gilbert and Basran highlight the evolutionary underpinnings of competitive behavior and leadership because without an understanding of human and social innate motivational systems and their algorithms, and the contexts that bring them to life, there may be little development of leadership and the social contexts which support well-being, social justice, and fairness. More importantly, society at large cannot afford to continue to endorse antisocial leaders. Gilbert and Basran urgently require development of leadership science that helps us to understand how to counteract antisocial appeal and support leaders with prosocial interests. Article four, authored by Basran et al. found that fear plays a driving role within antisocial leadership styles. Specifically, fears of compassion for others was a partial mediator of the relationship between insecure striving with an antisocial leadership style. Furthermore, the antisocial forms of leadership is driven by unaddressed threats and fears of rejection and compassion that require further research for greater promotion of prosocial leadership styles over antisocial leadership. Articles five and six authored by Hertel et al. and Abraham et al. both contribute to collective competition. Hertel et al. confirm perceived indispensability can be a significant (and often underestimated) motivator in occupational teams. Moreover, the findings suggest that building on social responsibility and concerns for others might be a more effective motivation strategy in teams than merely relying on individualistic concerns to successfully compete with others, while Abraham et al. findings suggest that team competition is observable and impactful at the micro-level of team interaction processes. Article seven from the Research Topic contributes to the understanding of the prisoner's dilemma game (PDG) as a tool to develop competences in dealing with the challenges of conflict management in organizational psychology (Bruno et al.). This is critically important for overcoming the individualistic stereotype in conflict representation by highlighting the interdependence of social interaction in human and social competition. Moreover, students' learning outcomes activates the development of organizational skills through PDG for improving conflict management in organizations. Lastly, in article eight of the collection, Ariza-Montes et al. reminds us that altruism and employees that find purpose in their work may lead to mutual cooperation in the workplace rather than human and social competition.

Vast gaps of human and social competition in organizational behavior remain understudied (Swab and Johnson, 2018). The editors hope that this Research Topic will enlighten and generate further research from readers that find human and social competition important to further explore and develop within the organizational psychology literature.

## Author Contributions

All authors listed have made a substantial, direct and intellectual contribution to the work, and approved it for publication.

### Conflict of Interest

The authors declare that the research was conducted in the absence of any commercial or financial relationships that could be construed as a potential conflict of interest.
